# Position in Sleeping

**Published:** 1876-06

**Authors:** 


### POSITION IN SLEEPING.

It is better to go to sleep on the right side, for then the stomach is very much in the position of a bottle turned upside down, and the contents are aided in passing out by gravitation. If one goes to sleep on the left side, the operation of emptying the stomach of its contents is more like drawing water from a well. After going to sleep, let the body take its own position. If you sleep on your back, especially soon after a hearty meal, the weight of the digestive organs, and that of the food, resting on the great vein of the body, near the back bone, compresses it, and arrests the flow of the blood more or less. If the arrest is partial, the sleep is disturbed, and there are unpleasant dreams. If the meal has been recent or hearty, the arrest is more decided, and the various sensations, such as falling over a precipice, or the pursuit of a wild beast, or other impending danger, and the desperate effort to get rid of it arouses us; that sends on the stagnating blood, and we wake in a fright, or trembling, or perspiration, or feeling of exhaustion, according to the degree of stagnation, and the length and strength of the effort made to escape the danger. But when we are not able to escape the danger, when we do fall over the precipice, when the tumbling building crushes us, what then? *That is Death!* That is the death of those of whom it is said, when found lifeless in their bed in the morning, "They were as well as they ever were the day before;" and often is it added, and *ate heartier than common!* This last, as a frequent cause of death to those who have gone to bed well to wake no more, we give merely as a private opinion. The possibility of its truth is enough to deter any rational man from a late and hearty meal. This we do know with certainty, that waking up in the night with painful diarrhœa, or cholera, or bilious colic, ending in death in a very short time, is properly traceable to a late large meal. The truly wise will take the safer side. For persons who eat three times a day, it is amply sufficient to make the last meal of cold bread and butter and a cup of some warm drink. No one can starve on it, while a perseverance in the habit soon begets a vigorous appetite for breakfast, so promising of a day of comfort.

